# Blood Metallothionein Transcript as a Biomarker for Metal Sensitivity: Low Blood Metallothionein Transcripts in Arsenicosis Patients from Guizhou, China

**DOI:** 10.1289/ehp.10035

**Published:** 2007-04-18

**Authors:** Jie Liu, Min-Liang Cheng, Qin Yang, Ke-Ren Shan, Jun Shen, Yushu Zhou, Xinjiang Zhang, Anna L. Dill, Michael P. Waalkes

**Affiliations:** 1 Inorganic Carcinogenesis Section, Laboratory of Comparative Carcinogenesis, National Cancer Institute at the National Institute of Environmental Health Sciences, National Institutes of Health, Department of Health and Human Services, Research Triangle Park, North Carolina, USA; 2 Guiyang Medical College, Guiyang, China; 3 Southwest Prefecture Center for Disease Control, Xingyi, Guizhou, China; 4 Zunyi Medical College, Zunyi, China

**Keywords:** arsenicosis patients, biomarker, blood, buccal cells, metallothionein

## Abstract

**Background:**

Because metallothionein (MT) is a metal-binding protein that protects against metal intoxication, it could be a biomarker for individual sensitivity to metal toxicity.

**Objective:**

We assessed the use of bloodborne *MT* transcript as a reflection of tissue MT levels and examined the potential role of MT in arsenic toxicity in an environmentally exposed human population.

**Method:**

Rodents were treated with zinc or nonmetallic MT inducers for 4 days, and the blood and tissues were collected for *MT* transcript analysis by real-time reverse transcriptase–polymerase chain reaction and MT protein determination by the cadmium–hemoglobin assay. Blood and buccal cell samples were collected from arsenicosis patients and healthy subjects residing in Guizhou, China, and total RNA was isolated for *MT* transcript analysis.

**Results:**

There was a positive correlation between blood *MT-1* and *MT-2* transcripts and corresponding hepatic or renal *MT* transcript levels in rats and mice. Furthermore, there was a positive correlation between blood *MT-1* and *MT-2* transcript and tissue MT protein levels in these animals. A positive correlation also occurred between human blood *MT* and buccal cell *MT* transcript levels. *MT-1A* and *MT-2A* were the major isoform transcripts in human blood and buccal cells, and significantly lower *MT* levels were seen in arsenicosis patients compared with healthy subjects.

**Conclusions:**

Blood *MT* transcript appears to be a useful biomarker of tissue MT levels. Arsenicosis patients in Guizhou show significantly lower *MT* transcript levels in blood, which may have predisposed this population to arsenic intoxication.

Metallothionein (MT) is a low-molecular-weight metal-binding protein (Kagi 1991). MT plays important roles in the detoxication of heavy metals, in the homeostasis of essential metals, and in the scavenging of free radicals (Kagi 1991; Klaassen et al. 1999). Moreover, MT expression can be greatly increased by exposure to a variety of agents and physiologic stimuli (Kagi 1991). This includes a variety of metallic agents that can activate MT gene expression. There are at least four mammalian major MT isoforms. The MT-1 and MT-2 isoforms are most widely expressed, whereas MT-3 is largely brain-specific and MT-4 is located mainly in stratified squamous epithelia (Cherian et al. 2003; Quaife et al. 1994).

Deficiency of MT clearly predisposes animals to metal intoxication and carcinogenesis. For example, MT-1/2–double knockout (MT-null) mice are more sensitive than wild-type mice to the toxicity of cadmium (Klaassen et al. 1999), mercury (Yoshida et al. 2004), arsenic (Liu et al. 2000), cisplatin (Satoh et al. 1997), and zinc and copper (Park et al. 2001). MT-null mice are also more susceptible to carcinogenic effects of lead (Waalkes et al. 2004), cisplatin (Waalkes et al. 2006), and cadmium (Waalkes MP et al., unpublished observation). Thus, the levels of MT appear to be a key factor in determining sensitivity to toxicity and carcinogenicity for various metals.

In humans for reasons that are not fully understood, there is great individual variation in MT expression (Allan et al. 2000; Wu et al. 2000). For example, in one study, MT protein levels in human liver without any pathology varied from 0 to 104 μg/g tissue (Sillevis Smitt et al. 1992). Various other authors have seen similar wide-ranging discrepancies in MT expression in human populations (Bem et al. 1988; Onosaka et al. 1986). It also appears that polymorphisms for human *MT-2A* gene can significantly affect MT expression (Kita et al. 2006). On the basis of these findings, we hypothesize that individuals with a low ability for MT expression may be susceptible to metal toxicity.

MT synthesis can be increased by arsenicals in mice or rats (Albores et al. 1992; Kreppel et al. 1993; Liu et al. 2000). Poor production of MT, as in MT-null mice, predisposes animals to the hepatotoxicity, nephrotoxicity, and lethality produced by inorganic arsenicals (Liu et al. 2000; Park et al. 2001). MT deficiency also can enhance the genotoxicity of methylated arsenicals (Jia et al. 2004). It is clear that arsenicals can bind to various MTs (Ngu and Stillman 2006; Toyama et al. 2002), including human MTs. However, little is known about any potential role of MT in arsenic toxicity in humans.

The use of MT measurement in readily accessible tissues, such as blood, as a bio-marker to predict expression levels of MT in other tissues has not been established in humans or rodents, and it is unknown if an individual poorly expressing MT in one tissue would show poor expression in other tissues. There are also difficulties in measuring very low levels of blood MT protein by the commonly used MT assays. Thus, in the present work, we initially determined whether blood *MT* transcripts, as measured by the highly sensitive and accurate real-time reverse transcriptase–polymerase chain reaction (RT-PCR) technique, correlate with tissue MT levels in rodents. We then examined *MT* expression in blood and buccal cells in a population from Guizhou, China, with clear dermal signs of chronic arsenicosis and elevated urinary arsenic levels (Liu et al. 2002) compared with a control population from the same district. The data provide evidence that blood *MT* transcript can be used as a readily available biomarker for individual ability to express MT in other tissues and that arsenicosis patients from Guizhou show lower expression of MT potentially as a predisposing factor.

## Materials and Methods

### Animals and treatment

Fischer 344 male rats weighing 200–220 g and male CD1 mice weighing 25–30 g were obtained from Charles River Laboratories (Wilmington, MA, USA). Animals were housed in facilities accredited by the American Association for the Accreditation of Laboratory Animal Care at the National Institute of Environmental Health Sciences (NIEHS) at 20–22°C with a 12-hr light/dark cycle for 1 week before treatment. Animals were allowed free access to food (Rodent Laboratory Chow #5002; Ralston Purina Co., St. Louis, MO, USA) and water. To help create varying levels of MT expression, groups (*n* = 4–6) of animals were given the metallic MT inducer zinc chloride (50, 100, or 200 mol/kg, sc), two nonmetallic MT inducers (ethanol, 2.5 g/kg, ig; or oleanolic acid, 50 mol/kg, sc), or equal volumes of saline as controls (2 mL/kg, sc) for consecutive 4 days. Twenty-four hours after the last dose, animals were killed by carbon dioxide asphyxiation to collect blood and tissues (liver and kidney). For correlative analysis, the individual data were grouped together by species (rat or mouse) to provide a wide range of tissue MT expression after different treatments. All procedures involving the use of laboratory animals were reviewed and approved by the Institutional Animal Care and Use Committee of the NIEHS. Animals were treated humanely and with regard for alleviation of suffering.

### Study population and sample collection

Adult patients were from an area of endemic arsenic intoxication in Guizhou province (Liu et al. 2002; Lu et al. 2001) and were selected for this study on the basis of arsenic exposure history, including elevated urinary arsenic excretion, and arsenic-induced skin lesions (hyperkeratosis, hyperpigmentation, etc.), as well as other clinical symptomology of chronic arsenic intoxication (Yang et al. 2005; Zhang et al. 2006; Zhao et al. 2005). Controls were from the same province and were selected for an absence of signs or symptoms of arsenic intoxication. The arsenicosis patients are exposed to the burning of high-arsenic–containing coal for heating and drying food items and would have a complex exposure by mixed routes (Liu et al. 2002). In addition, they would be exposed to other components of coal. Ethanol use and smoking were not assessed in this group. Venous blood (0.5–0.7 mL) was collected directly in a tube containing 0.75 mL Trizol. For buccal cell collection, the mouth was rinsed with water, followed by brushing with tooth brush and rinsing with saline that was then collected into a 50-mL tube. The saline rinse was then centrifuged to pellet cells, and the saline was decanted off. Trizol (1 mL) was added to the pellet and the material was resuspended, then transferred to a 1.5-mL tube. For blood cell MT analysis, 48 arsenicosis patients (26 males and 22 females) and 48 healthy subjects from the same district (23 males and 25 females) were selected. For buccal cell analysis, 44 arsenicosis patients were compared with 12 healthy subjects. This study has complied with all applicable requirements of the U.S. and/or international regulations (including approval from the Guizhou Health Department), and human participants gave written informed consent before the study. The U.S. portion of this study was performed under a confidentiality agreement approved by the Office of Human Subjects Research, National Cancer Institute.

### RNA extraction and real-time RT-PCR

Total RNA was extracted from rodent blood or tissues, or from human blood or buccal cells with Trizol reagent (Invitrogen, Carlsbad, CA, USA), followed by purification with RNeasy columns (Qiagen, Valencia, CA, USA). The purity and quantity of RNA were determined with an ultraviolet spectrophotometer (Shimadzu Scientific Instruments, Columbia, MD, USA) with the A260/A280 ratio > 1.7. All the RNA isolation procedures were performed at Guiyang Medical College. Purified RNA was reverse transcribed with MuLV (murine leukemia virus) reverse transcriptase and oligo-dT primers. The forward and reverse primers for selected genes were designed using Primer Express software (Applied Biosystems, Foster City, CA, USA) and are listed in Table 1. The SYBR Green Master Mix (Applied Biosystems) was used for real-time RT-PCR analysis. Differences in gene expression were calculated using threshold cycle (Ct) values, which were normalized to β*-actin*. The basal level for β*-actin* expression was consistently about 21 ± 1.5 Ct in both arsenic-exposed and normal groups and thus was used as the housekeeping gene. The arbitrary assigned Ct value was subtracted for all *MT* isoforms to generate their relative expression levels.

### MT protein determination

Rodent liver and kidney were homogenized in 10 mM Tris–HCl buffer (1:5, wt:vol), followed by centrifugation at 20,000 × *g* for 10 min. MT protein concentrations in the cytosol were then determined by the cadmium–hemoglobin assay (Eaton and Toal 1982).

### Statistical analysis

Means and standard errors for each *MT* isoform and subgroup were calculated. Two-way Student’s *t*-tests were performed with the level of significance set at *p* < 0.05. Linear (Pearson) correlations were used to determine statistical significance of correlations between blood *MT* transcript and tissue MT transcript or protein levels. The level of significance was set at *p* < 0.05.

## Results

### Correlation of blood MT transcript with tissue MT transcript in animals

**Figure 1:**
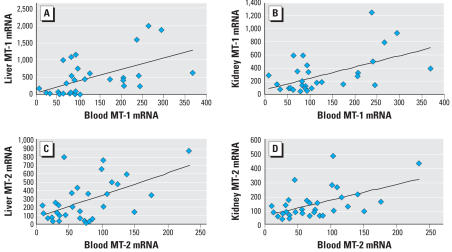
Correlation analysis of blood transcripts with tissue *MT* transcripts in rats. (*A*) Correlation of blood *MT-1* transcript with hepatic *MT-1* transcript. (*B*) Correlation of blood *MT-1* transcript with renal *MT-1* transcript. (*C*) Correlation of blood *MT-2* transcript with hepatic *MT-2* transcript. (*D*) Correlation of blood *MT-2* transcript with renal *MT-2* transcript. Rats (*n* = 32) were treated with various MT inducers as detailed in “Materials and Methods,” and *MT* transcript levels were quantified by real-time RT-PCR.

To determine whether *MT* transcript levels in blood would be reflective of MT in other tissues, rats and mice were treated with various inducers of MT (zinc, ethanol, oleanolic acid) or saline for 4 days to create varying MT expression levels. *MT* transcript or protein levels were determined 24 hr after the last treatment. Individual data were grouped together for correlative analysis, and correlations were tested using individual values. The correlation analysis between blood *MT-1* and *MT-2* transcript and corresponding liver or kidney transcript in rats is shown in Figure 1. There were significant correlations between *MT-1* transcript in the blood and in the liver (Figure 1A; *p* < 0.001, *r* = 0.540) and between blood and the kidney (Figure 1B; *p* < 0.002, *r* = 0.536). Similarly, significant correlations occurred between *MT-2* transcript in blood and liver (Figure 1C; *p* < 0.001, *r* = 0.546) and between the blood and kidney (Figure 1D; *p* < 0.01, *r* = 0.519). Significant correlations between blood *MT* transcripts and tissue *MT* transcripts were also evident in mice (data not shown)

### Correlation of animal tissue MT protein with MT transcripts

**Figure 2:**
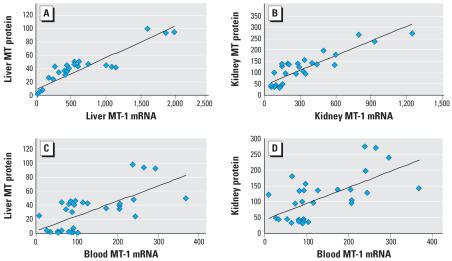
Correlation analysis of (*A*) hepatic MT protein with hepatic *MT-1* mRNA levels; (*B*), renal MT protein with renal *MT-1* mRNA levels; (*C*) hepatic MT protein with blood *MT-1* mRNA levels; and (*D*) renal MT protein with blood *MT-1* mRNA levels. Rats (*n* = 32) were treated with various MT inducers as detailed in “Materials and Methods”; MT transcript levels were quantified by real-time RT-PCR, and MT protein was determined by the cadmium–hemoglobin assay.

The correlation between hepatic or renal MT protein levels with concordant MT transcript levels in the respective tissue in rats is shown in Figure 2. There was a strong correlation between individual hepatic MT protein level and hepatic *MT-1* transcript (Figure 2A; *p* < 0.001). Similarly, renal MT protein levels correlated with renal *MT-1* transcript (Figure 2B; *p* < 0.001). Highly significant correlations also occurred between MT protein and *MT-2* transcript (not shown) in both the liver (*p* < 0.001, *r* = 0.924) and kidney (*p* < 0.001, *r* = 0.883). To be useful as an indicator of individual MT expression, and thus as a potential biomarker for sensitivity to metal intoxication, MT expression measured in a readily accessible sample (i.e., blood) would have to correlate with individual tissue protein levels. Thus, the correlation between liver or kidney MT protein and blood MT transcript was tested in rats. The results indicate that a strong correlation occurred between blood *MT-1* transcript levels and hepatic MT protein (Figure 2C; *p* < 0.001, *r* = 0.672), and renal MT protein (Figure 2D; *p* < 0.001, *r* = 0.641). Highly significant correlations also occurred between tissue MT protein and blood *MT-2* transcript in both the liver (*p* < 0.001) and kidney (*p* < 0.001) (data not shown). Thus, it appears blood MT transcript levels are correlated with tissue MT protein, at least for the liver and kidney. Significant correlations between blood *MT* transcripts and tissue *MT* transcript or protein levels were also evident in mice (data not shown)

### MT isoforms in human blood

**Figure 3:**
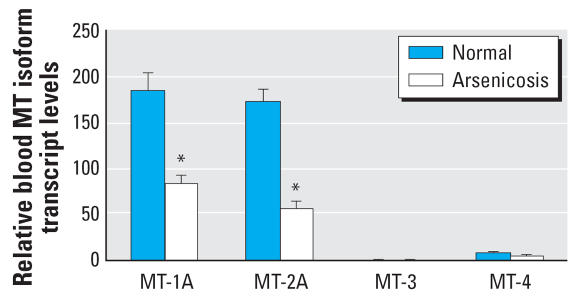
The expression of human *MT* isoforms in blood cells of arsenic-exposed patients (*n* = 48) and healthy subjects (*n* = 48) in Guizhou, China. Total RNA was extracted, purified, reverse-transcripted, and subjected to SYBR Green real-time quantitative RT-PCR with *MT* isoform primers. Data are mean ± SE. *Significantly different from healthy controls at *p* < 0.05.

Human blood contains transcripts of at least 10 MT isoforms (Chang et al. 2006; Vandeghinste et al. 2000). Figure 3 illustrates the relative transcript levels of *MT-1A, MT-2A, MT-3,* and *MT-4* in blood of arsenic-intoxicated patients compared with healthy subjects. The transcript levels for the major MT isoforms *MT-1A* and *MT-2A* were significantly lower (*p* < 0.05) in arsenicosis patients than in healthy subjects. In addition, regardless of arsenic exposure, higher levels of MT transcripts were seen with the *MT-1A* and *MT-2A* isoforms relative to other isoforms, whereas expression ranged from low for *MT-4* to barely detectable for *MT-3* in the present study.

### Blood MT isoforms in male and female subjects

**Figure 4:**
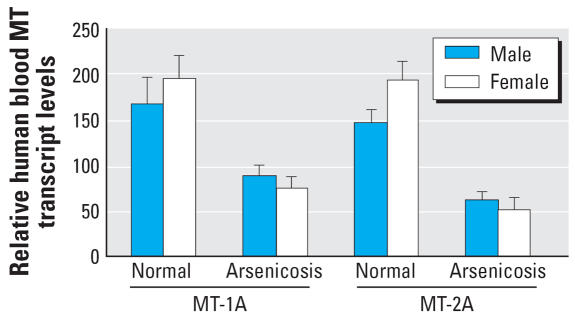
The sex difference in human *MT-1A* and *MT-2A* expression in blood cells of arsenicosis patients (26 males and 22 females) and healthy subjects (23 males and 25 females) in Guizhou, China. Total RNA was extracted, purified, reverse-transcripted, and subjected to SYBR Green real-time quantitative RT-PCR with *MT* isoform primers. Data are mean ± SE.

Some evidence indicates there may be sex-dependent sensitivity to arsenic toxicity (Ahmad et al. 1999; Liu et al. 2000; Waalkes et al. 2003) in rodents. Thus, the expression of major *MT* isoforms was compared in males and females (Figure 4). No major differences in *MT-1A* and *MT-2A* isoform expressions between males and females were evident either from the normal healthy subjects or from arsenicosis patients.

### MT isoforms in human buccal cells

**Figure 5:**
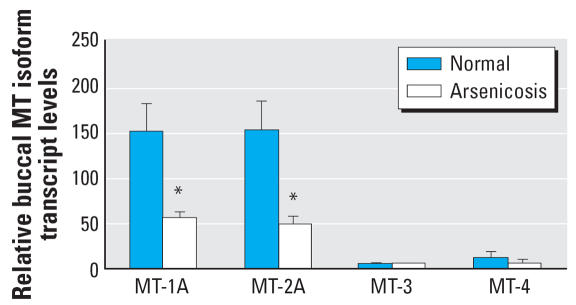
The expression of human *MT* isoforms in buccal cells of arsenic-exposed patients (*n* = 44) and healthy subjects (*n* = 12) in Guizhou, China. Total RNA was extracted, purified, reverse-transcripted, and subjected to SYBR Green real-time quantitative RT-PCR with *MT* isoform primers. Data are mean ± SE. *Significantly different from controls at *p* < 0.05.

MT expression in human buccal cells has not been previously reported. In the present study (Figure 5), as with transcript in blood, the *MT-1A* and *MT-2A* isoforms in the buccal cells were predominant. The expressions of *MT-3* and *MT-4* isoforms were low. Consistent with blood transcript levels, the relative expressions of *MT-1A* and *MT-2A* were significantly lower in arsenicosis patients than in healthy subjects. The expressions of *MT-3* and *MT-4* isoforms were too low for valid comparison.

### *Correlation of human* MT *transcript in blood and buccal cells.*

**Figure 6:**
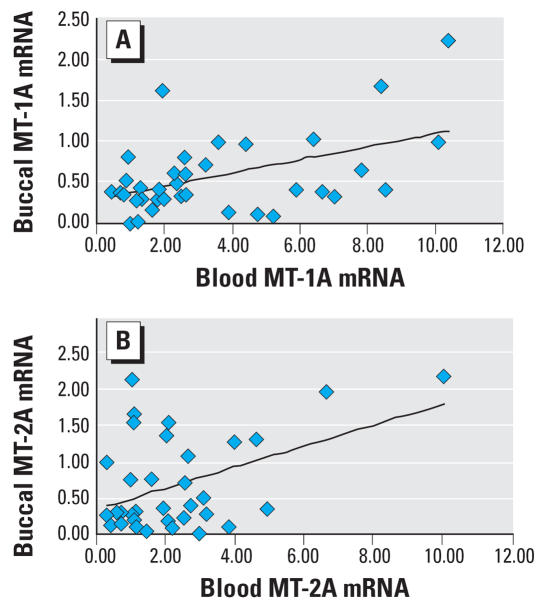
Correlation analysis of *MT-1A* (*A*) and *MT-2A* (*B*) expression between blood cells and buccal cells of arsenicosis patients from Guizhou, China (*n* = 36). Sample collection and *MT* determination by real-time RT-PCR are described in detail in “Materials and Methods.”

Human *MT-1A* and *MT-2A* transcript levels in blood and buccal cells from 36 human subjects were used for correlation analysis (Figure 6). There was a highly significant correlation between individual blood *MT-1A* transcript and buccal cell *MT-1A* transcript levels (Figure 6A; *p* < 0.01, *r* = 0.487). Similarly, individual blood *MT-2A* transcript was strongly correlated (*p* < 0.01, *r* = 0.434) with buccal cell *MT-2A* (Figure 6B).

## Discussion

The present study demonstrates the potential utility of blood *MT* transcripts as a biomarker for tissue MT levels in rodents and in humans. A clear correlation between blood *MT* transcript levels and hepatic and renal *MT* transcripts is evident in rats and mice, and a clear correlation of blood MT transcripts with tissue MT protein levels is also evident in these animals. Furthermore, there is a correlation between human blood and buccal cell *MT* transcript levels in humans. Thus, blood *MT* transcripts appear to be a useful biomarker for target tissue MT levels.

In arsenicosis patients, distinctly lower levels of blood *MT* isoform transcripts were consistently observed. The transcript levels of *MT* isoforms in buccal cells of arsenicosis patients were also significantly lower than in healthy subjects. For example, the major isoforms of *MT-1A* and *MT-2A* transcript levels in arsenicosis patients were at least 65% (blood) and 74% (buccal cells) lower than that in control. These *MT* isoform levels were independent of sex. There are several factors that may be the basis for the lower *MT* expression seen in the arsenicosis patients in the present study. First, lower *MT* expression may have been a predisposing factor to arsenic toxicity. In this regard, not all people living in this area of endemic arsenicosis show overt signs of arsenic intoxication (Liu et al. 2002; Yang et al. 2006; Zhang et al. 2006), so some differences in sensitivity might be expected. Differences between the control population and the arsenicosis population could contribute variation in MT, and, for example, smoking and/or ethanol consumption were not assessed in the present study. Both ethanol and cigarette smoke condensate will increase MT expression in animals or in cultured cells (Chen et al. 2005; Klaassen et al. 1999). Finally, the arsenicosis patients are also exposed to various components of coal combustion, and this may have altered MT expression. Thus, there are various factors that may have contributed to the finding of low MT in association with human arseni-cosis, and further work will be required to define it as a predispositional factor.

Erythrocytes in the blood appear unable to synthesize proteins, including MT, and thus *MT* transcript in the blood likely comes from monocytes and lymphocytes (Koizumi et al. 1987) or cellular turnover of internal tissues. In rodents, proerythroblasts have been hypothesized as the site of MT biosynthesis, which may contribute to MT present in mature erythrocytes (Tanaka et al. 1985). In any event, the commonly used cadmium–hemoglobin assay is unable to reliably detect MT protein in the blood, probably because of abundant sample hemoglobin and interference by other blood components. Thus, isolated leukocytes have been used to evaluate MT production after *in vitro* exposure to various MT inducers (Allan et al. 2000; Yurkow and DeCoste 1999) but have not been used to evaluate MT in other tissues. The results of the present study clearly indicate that blood *MT* transcript are predictive of tissue *MT* gene expression in rodents, and human blood *MT* transcript levels are correlated with buccal cell *MT* transcript levels in the present study. Thus, blood *MT* transcript determinations could provide insight into MT levels in human tissues and could be used to identify susceptible populations.

In animal studies, arsenic effectively activates *MT* gene expression (Albores et al. 1992; Kreppel et al. 1993; Liu et al. 2000). The induction of MT by arsenicals can be envisioned, at least in part, as an adaptive response to overcome toxic insult from this metalloid. For example, MT-null mice are more clearly sensitive than wild-type mice to arsenic-induced acute lethality (Park et al. 2001) and chronic hepatotoxicity or nephrotoxicity (Liu et al. 2000). In addition, poor production of MT makes mice more susceptible to dimethylarsinic acid–induced DNA strand breaks in peripheral blood cells (Jia et al. 2004). These data support a protective role of MT against arsenical toxicity, at least in part, by binding arsenic to MT and thus sequestering arsenic from critical cellular organelles (Ngu and Stillman 2006; Toyama et al. 2002). MT-null mice were more susceptible to carcinogenic effects of other metal compounds such as lead and cisplatin (Waalkes et al. 2004, 2006). Therefore, tissue MT levels in rodents appear to dictate the sensitivity to arsenic intoxication, with lower levels enhancing susceptibility. It is noteworthy that MT levels were low in the arsenic-exposed population studied in the present work even though arsenic can effectively activate MT synthesis (Albores et al. 1992; Kreppel et al. 1993; Liu et al. 2000). Thus, one valid interpretation of these human data is that poor MT expression may have predisposed these patients from Guizhou to arsenic intoxication. It is important to note that although individuals can be exposed to similar arsenic levels, the symptoms of arsenic intoxication can vary widely in the population from Guizhou (Liu et al. 2002; Yang et al. 2005; Zhao et al. 2005; Zhou et al. 2000), and the present group was selected based on overt evidence of arsenicosis.

Although MT can be induced easily by a variety of stimuli, low MT expression has also been reported in a variety of tumors (Jacob et al. 2002). For example, poor MT expression occurs with human and murine hepatocellular tumors compared with tumor-surrounding tissues (Lu et al. 2003; Waalkes et al. 1996). Relative expression of MT is, however, dependent on the type of tumor, tissue, and stage of the disease (Cherian et al. 2003; Jin et al. 2004). For example, many human tumors show relatively high expression of MT. So it does not appear that suppression of MT expression is a general characteristic of oncogenesis or carcinogen exposure. In our recent transplacental arsenic carcinogenesis studies, hepatic MT was initially increased immediately after arsenic exposure ended on gestation day 18 and at birth, but by 104 weeks of age, the expression of *MT* in arsenic-induced liver tumors or in normal surrounding liver is only half that of the controls (Liu et al. 2006). This might indicate that there was an initial MT induction by arsenic that was lost when arsenic exposure was discontinued. Not all arsenic-treated mice developed tumors (Waalkes et al. 2003), and MT expression was not assessed in mice without arsenic-induced tumors. In any event, the decreased *MT* expression in blood and buccal cells of the arsenicosis patients in the present study occurred during continuous exposure to arsenic, and the data indicating arsenic can induce MT (Albores et al. 1992; Kreppel et al. 1993; Liu et al. 2000) make it difficult to envision reduced MT as an effect of arsenic exposure. Expression polymorphism of MT exists in humans (Kita et al. 2006; Wu et al. 2000) that could potentially predispose populations to arsenic toxicity and carcinogenesis. Further work is required to define the role of MT in arsenic toxicity and carcinogenicity.

Low expression of *MT* in arsenicosis patients could also be due to alterations in cell proliferation, or cell differentiation, such as the events seen during tumor progression (Cherian et al. 2003), or due to indirect methylation of the *MT* genes (Jacob et al. 2002). *MT* gene hypermethylation is associated with reduced expression in various human or rodent tissues and cells (Compere and Palmiter 1983; Deng et al. 2003; Huang et al. 2003; Jacob et al. 2002). Chemical or physical alterations in *MT* gene methylation significantly alter expression potential (Compere and Palmiter 1983; Huang et al. 2003; Jacob et al. 2002). Whether the decreased *MT* isoform expression in blood and buccal cells of arsenic-exposed patients is associated with differences in *MT* gene methylation warrants further investigation.

This study demonstrated for the first time that the expression of *MT* isoforms can be analyzed in buccal cells, and that the results from buccal cells were consistent with blood samples. Buccal cells have been used to detect arsenic-induced DNA damage (micronuclei assay and apoptosis) in arsenic-exposed populations from Inner Mongolia, China (Tian et al. 2001), and from Chile (Martinez et al. 2005). The collection of buccal cells is more practical for examining arsenic-induced aberrant gene expression than most other tissues. In this regard, the buccal cell gene expression analysis of the same arsenic-exposed population from Guizhou found increased *TP53* gene expression, but no alterations in α-feto-protein expression (Yang et al. 2005). Thus, gene expression analysis with buccal cells could have potential use as a noninvasive measure of arsenic-induced aberrant gene expression and toxicity.

In summary, the current study indicates blood *MT* expression is indicative of tissue MT expression in rats and mice and that poor expression of MT in blood and buccal cells is associated with arsenicosis in humans. Limited production of MT could be a susceptiblity factor in arsenic toxicity, and, perhaps, arsenic carcinogenesis.

## Figures and Tables

**Table 1:** Sequence of primers used in SYBR Green real-time PCR analysis.

Gene symbol	Accession no.^a^	Forward	Reverse
β*-actin* (human)	X00351	ACTGGAACGGTGAAGGTGACA	ATGGCAAGGGACTTCCTGTAAC
*MT-1A* (human)	NM_005946	CTCGAAATGGACCCCAACTG	CAGCCCTGGGCACACTTG
*MT-2A* (human)	NM_005953	GTGCCCAAGGCTGCATCT	GGTCACGGTCAGGGTTGTACA
*MT-3* (human)	NM_005954	AGTGCGAGGGATGCAAATG	GCCTTTGCACACACAGTCCTT
*MT-4* (human)	U07807	TCCAGGCCTCATGTGATTCAC	CCCTCTTGGCTAGGCACAGT
β*-actin* (rat)	V01217	TCCTCCTGAGCGCAAGTACTCT	GCTCAGTAACAGTCCGCCTAGAA
*MT-1* (rat)	NM_138826	TGTGCCTGAAGTGACGAACAG	TTCACATGCTCGGTAGAAAACG
*MT-2* (rat)	M11794	GGGAACTGGGCAGGAATAACA	CAGCCTCAAGCCAGGATGTC
β*-actin* (mouse)	M12481	GGCCAACCGTGAAAAGATGA	CAGCCTGGATGGCTACGTACA
*MT-1* (mouse)	BC027262	AATGTGCCCAGGGCTGTGT	GCTGGGTTGGTCCGATACTATT
*MT-2* (mouse)	NM_008630	TGTGCCTCCGATGGATCCT	GCAGCCCTGGGAGCACTT

a From the National Center for Biotechnology Information, Entrez Nucleotide Database (http://www.ncbi.nlm.nih.gov/entrez/query.fcgi).
